# Colonoscopic finding in children with lower gastrointestinal complaints

**DOI:** 10.1002/jgh3.12991

**Published:** 2023-11-08

**Authors:** Shinwari Abdullah Jan, Ghayour Ajmal, Zaheer Naimatullah

**Affiliations:** ^1^ Medical Faculty Nangarhar University Jalalabad Afghanistan; ^2^ Stomatology Faculty Kunduz University Kunduz Afghanistan

**Keywords:** colonoscopic findings, colonoscopy procedure, colorectal polyp, lower gastrointestinal complaints

## Abstract

**Background and Aim:**

Colonoscopy is an important tool for the diagnosis and treatment of lower gastrointestinal (LGI) diseases in both children and adults. This study describes an endoscopic profile of children at the Shinnwari Gastroenterology Diagnostic Clinic in Jalalabad, Afghanistan.

**Methods:**

This is a cross‐sectional descriptive study conducted in children ≤16 years, taken from recorded colonoscopy reports from 1 January 2021 to 30 December 2022.

**Results:**

Of the 672 colonoscopy procedures, 250 were diagnostic in children (7.41 years median age; 2.5:1 male/female ratio) without serious complications. Abnormal findings were recorded in 201 (81.2%) procedures; the most common presentation was hematochezia, which was higher in 5–8‐year‐olds. More frequent findings were colorectal polyps (50%), infection (16.4%), internal hemorrhoid (IH; 10%), and inflammatory bowel disease (IBD; 1.2%). Incidences of colorectal polyps were higher in those aged <9 years (37.2% *vs* 12.8%; *P* < 0.001). Conversely, internal IH and IBD tended to be higher in older children (aged ≥9 years) (IH: 6.8% *vs* 3.2%; *P* < 0.005; IBD: 1.2% *vs* 0%; *P* < 0.02). Colonoscopy procedures were completed without major complications.

**Conclusion:**

Colonoscopy is an important and safe procedure for the diagnosis of LGI compliants, especially hematochezia, which is frequently accompanied by colorectal polyps.

## Graphical abstract

Abnormal findings recorded in 201(81.2%) procedures; the most common presentation was hematochezia that was more in 5–8 years. More frequent findings were Colo‐rectal polyps (50%), infection (16.4%), internal hemorrhoid (10%) and IBD (1.2%). Rate of Colo‐rectal polyps were higher in comparison with in those aged ≥9 years old (37.2% vs. 12.8%; P < 0.001). Conversely, internal hemorrhoid and inflammatory bowel disease tended to be found very often in the older ≥9 years (6.8% vs. 3.2%; P < 0.005, IBD: 1.2% vs. 0%; P < 0.02).

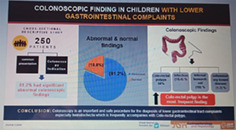

## Introduction

Children frequently complain about gastrointestinal disorders, which present with different clinical states. Diagnosis is made through clinical and laboratory examinations. In many instances, for definite diagnosis, radiography, direct visualization, and microscopic techniques are used. Colonoscopy is being used worldwide for assessing lower gastrointestinal (LGI) bleeding, colonic diseases, and colorectal polyps, as well as for therapy, for many years.

From late 1970s, rapid developments have taken place in the field of colonoscopy for the diagnosis and treatment of gastrointestinal disorders in children, particularly in flexible endoscopy.^1^ Nowadays, modern techniques are widely used for diagnosis and treatment, which enable endoscopy to be performed in all age groups, including newborns. In the past two decades, safety and effectiveness of diagnostic and therapeutic colonoscopy in adults have been well demonstrated. Indications of colonoscopy in children are similar to those in adult patients. The most frequent symptoms are hematochezia, unexplained diarrhea, and unrelieved abdominal pain.^1^, ^2^ A large multicenter study in the United States has identified rectal bleeding, abdominal pain, and diarrhea as the most occurring indications for pediatric colonoscopy.^3^ In addition, chronic colitis, suspected inflammatory bowel disorders, malignancies, dilatation of colonic strictures, and removal of foreign bodies are other indications of colonoscopy.^4^ The main difficulties for performing colonoscopy in children is poor compliance with bowel cleansing, noncooperation during the procedure, and the high level of technical challenge. Compared to adult colonoscopy, pediatric colonoscopy has slightly higher risk of severe complications, which might also restrict its use in pediatric patients.^1^

Colonoscopic findings in infants and young children are inflammatory bowel disease (IBD), eosinophilic gastrointestinal disorders (EGIDs), and polyps/polyposis. Instances of IBD have been increasing over the past decade in children in southern China.^5^, ^6^ Worldwide, the increasing trend of IBD and EGIDs has escalated the role of colonoscopy in infants and younger children.^6^ IBS is reported in approximately 20–30% of children.^7^ Adenomatous changes in juvenile polyps have also been reported in recent studies, which indicate their neoplastic potential.^8^, ^9^ Colorectal polyps are the frequent findings of pediatric colonoscopy, which usually present with painless bleeding and generally are considered benign.^1^ Approximately 1% prevalence of colorectal polyps is reported in children of school‐going age, of which 90% are juvenile types. Two in 100 000 cases of IBD are reported, which are largely found in the 10–19‐year age group.^10^

Colorectal diseases are common throughout the world, but the spectrum of diagnoses in Afghanistan has remained largely unexplored. The aim of this study is to describe the indications of colonoscopy and colonoscopic findings in ≤16‐year‐old children in Jalalabad, Afghanistan.

Colonoscopy in children is a safe and effective procedure.^5^ Early diagnosis of LGI diseases, especially polyps and IBD, leads to correct treatment, prevents further severity, and ultimately can significantly improve the patients' quality of life and overall health. Therefore, in children colonoscopy is the tool of choice both for early lesion detection and as an effective therapy to treat lesions and remove polyps.

## Materials and methods

### Study design and participants

This is a cross‐sectional descriptive study, and potentially eligible participants were ≤16‐year‐old children with colonoscopy reports, who were referred by hospitals, pediatricians, pediatric surgeons, and clinicians for colonoscopy procedure to Shinwari Gastroenterology Diagnostic Clinic from 1 January 2021 to 30 December 2022, in Jalalabad, Afghanistan. The colonoscopy reports of all those children were included in the study who were aged ≤16 years and had any one of the following complaints: hematochezia, chronic diarrhea, unexplained lower abdominal pain, unexplained anemia, and anal mass protrusion. Children who were diagnosed as suffering from dysentery (known cause with routine lab exams such as amebiases or giardiasis) and had reports of repeated follow‐up colonoscopy for previous known diseases were excluded. All referred patients were first screened by an endoscopist according to the inclusion and exclusion criteria and then selected for colonoscopy procedure. The procedure was explained to the children's parents and consent was taken before the procedure.

### Shinwari Diagnostic Gastroenterology and Endoscopy Clinic

This is the only GI tract endoscopic diagnostic center in the entire eastern region of Afghanistan, in Jalalabad city, which has been performing colonoscopy procedures along with upper endoscopy of the GI tube for the last few years. Earlier, these patients used to be referred to Kabul or the neighboring countries. All these children with LGI complaints are now being referred by hospitals and private clinicians in the eastern region for gastrointestinal endoscopy procedures, which are performed by an internal medical specialist and expert gastrointestinal endoscopist, Dr. Abdullah Jan Shinwari.

Bowel preparation and cleaning in 1–5‐year‐old children were performed with 1.5 g/kg/day PEG 3350 without electrolytes in three divided doses for 2 days. They were advised liquid diet 24 h prior the procedure and one large bowel wash with normal saline enema (5–10 mL/kg). PEG 3350 without electrolytes was given 1.5 g/kg/day in three divided doses plus bisacodyl 5 mg (for ≤23 kg) or 10 mg (for >23 kg) for 2 days to the 5–10‐year‐old children and liquid diet was advised 24 h prior the procedure, whereas 11–16‐year‐old children were given 3 L PEG 3350 with electrolytes in 4 h for 2 days with one large bowel washing/cleaning phosphate enema. Colonoscopy of 1–4‐year‐old children was performed under general anesthesia, supervised closely during and after the procedure by an anesthetist. Children ≥5 years received midazolam (2 mg/kg) and IM injection of pethidine (1 mg/kg), chlorpromazine (0.5 mg/kg), and promethazine (0.5 mg/kg) as required. Colonoscopy was performed with Olympus CLV pediatric and adult colonoscopes. Each procedure was recorded as soft and hard copies in Microsoft Word. Essential demographic information such as name, age, sex, and address, as well as indication and detailed colonoscopic findings, are documented in a report form. These reports are collected and entered into a computerized information bank and analyzed by SPSS (version 16) by descriptive statistics as needed.

### Data management and statistical analysis

All the data regarding age, sex, colonoscopic indications, colonoscopic findings, and colorectal polyps as well as their number, type, and location were obtained from the colonoscopy reports. Based on this information, data variables and dataset were formed in SPSS 2016. Data on colonoscopic indication and findings are shown descriptively for four age groups: group 1 (1–4 year), group 2 (5–8 years), group 3 (9–12 years), and group 4 (13–16 years). Associations of LGI manifestation with colonoscopic finding and rectal bleeding with colorectal polyps are described. Data on patients with colorectal polyps are shown, with significance level set at *P* < 0.05.

### Ethics statement

Our study protocol was approved by the research and ethics committees of the Nangarhar Medical Faculty (NMF REC 016). Patient information was anonymously handled.

## Results

Out of a total of 672 colonoscopy examinations, 250 procedures were performed on patients aged ≤16 years, with a mean age of 7.41 years and a male/female ratio of 2.5:1; 67.6% (169) were carried out in patients aged <9 years. The cecal intubation rate was 67.2% (168) with 50% success rate in ileal intubation; 46 (18.4%) and 36 (14.4%) patients were examined up to hepatic and splenic flexure, respectively. Additionally, 66 (26.4%) and 102 (40.8%) procedures were performed under general anesthesia and intravenous sedation, respectively.

Two‐hundred and one (80.4%), 22 (8.8%), 16 (6.4%), 3 (1.2%), 5 (2%), and 3 (1.2%) children presented with rectal bleeding, constipation, diarrhea, unexplained anemia, lower abdominal pain, and anal mass protrusion, respectively. Rectal bleeding was the common indication in all age groups and was more frequent in 5–8‐year‐olds (87; 34.8%) and 1–4‐year‐olds (40; 16%).

Abnormal macroscopic findings were found in 81.2% of patients: the most frequent findings were colorectal polyps (50%), infections (16.4%), and hemorrhoid (10%). IBD (1.2%), polyposis (0.8%), mega colon (0.8%), rectal growth (0.8%), anal stricture with mucosal growth lesion (0.4%), and solitary rectal ulcer (0.4%) were less frequent. Colorectal polyps were occurring mostly in the 5–8‐year age groups, whereas internal hemorrhoid (IH) and IBD were more frequent in late age groups (Table 1).

**Table 1:** Frequency of colonoscopic finding in the cases by age group.

	*n* (%)						
Colonoscopic finding	G1 (1–4 years) (*n* = 66)	G2 (5–8 years) (*n* = 103)	G3 (9–12 years) (*n* = 48)	G4 (13–16 years) (*n* = 33)	Total	*χ* ^2^	*P‐*value
Polyps	27	66	22	10	125 (50)	637	<0.001*
Infection	14	15	7	5	41 (16.4)		
Normal appearance	19	15	9	4	47 (18.8)		
Internal hemorrhoid	3	5	8	9	25 (10)		
IBD	0	0	1	2	3 (1.2)		
Polyposis	0	1	1	0	2 (0.8)		
Mega colon	2	0	0	0	2 (0.8)		
Rectal growth	0	0	0	2	2 (0.8)		
Anal canal stricture with mucosal growth lesion	0	0	0	1	1 (0.4)		
Rectal prolapse	1	0	0	0	1 (0.4)		
Solitary rectal ulcer	0	1	0	0	1 (0.4)		

* Highly significant.

IBD, inflammatory bowel disease.

Clinical presentations were compared with colonoscopic findings, in which hematochezia was the most common presentation and was more common in patients with polyps, internal hemorrhoids, and infections. There were no serious complications during the procedure, because of careful and conscientious procedure, good bowel cleansing, sufficient risk evaluation preceding the procedure, and appropriate sedation or anesthesia by the endoscopist.

According to the data, hematochezia was the most frequent primary indication (*n* = 201; 80.4%). Diagnoses found that 1–16‐year‐old children were showing hematochezia including polyps (*n* = 125; 62%), infection (*n* = 33; 16.4%), IH (*n* = 22; 10.9%), polyposis (*n* = 2; 1%), ulcerative colitis (*n* = 2; 1%), polypoidal growth lesion (*n* = 2; 1%), anal canal stricture with mucosal growth lesion (*n* = 1; 0.5%), and solitary rectal ulcer (*n* = 1; 0.5%). We compared the nature of disease distribution in children aged <9 years and those aged 9–16 years. The most prevalent diagnosis was polyps, and the frequency was higher in the former group than in those aged 9–16 years (37.2% *vs* 12.8%; *P* < 0.001) (Table 2). Conversely, IH and IBD tended to be more frequent in the older age group (IH: 6.8% *vs* 3.2%; *P* < 0.001, IBD: 1.2% *vs* 0%; *P* = 0.02).

**Table 2:** Colonoscopic finding in the cases of rectal bleeding

Findings	*n* (%)	*χ* ^2^	*P‐*value
Polyp	125 (62.2)	39	<0.001*
Infection	33 (16.4)		
Internal hemorrhoid	22 (10.9)		
Normal appearance	13 (6.5)		
Polyposis	2 (1.0)		
Ulcerative colitis	2 (1.0)		
Polypoidal growth	2 (1.0)		
Anal canal stricture with mucosal growth lesion	1 (0.5)		
Solitary rectal ulcer	1 (0.5)		
Total	201 (100)		

* Highly significant.

**Figure 1:**
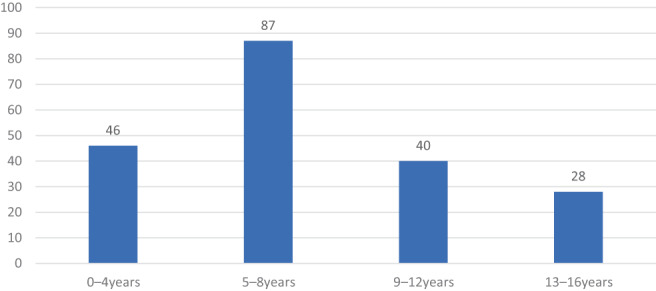
Rectal bleeding cases according to age groups.

Hematochezia was the most frequent presentation in 5–8‐year‐olds (87, 34.8%) and 0–4‐year‐olds (46, 18.4%) but was less frequent in 13–16‐year‐olds (28, 11.2%) (Fig. 1).

**Figure 2:**
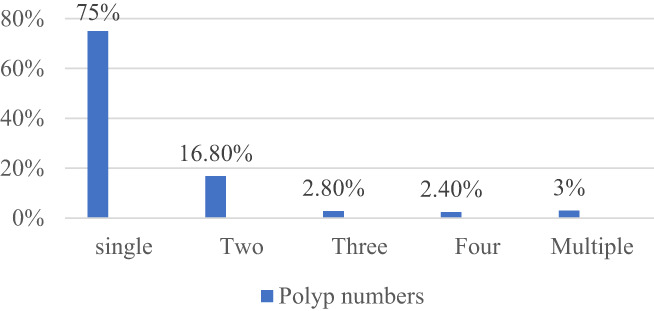
Number of polyps in patients.

In terms of number, one or two polyps were more frequent, but three or more polyps were less frequent (Fig. 2).

**Figure 3:**
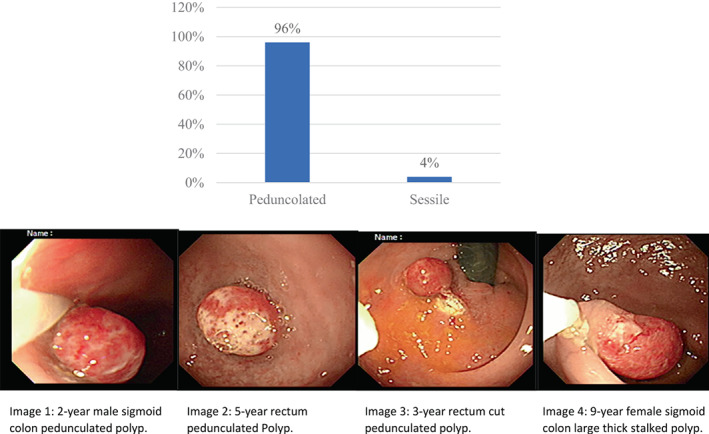
Morphological types of colorectal polyps and images.

Morphologically, pedunculated types were more, but there were a few polyps with sessile and broad base (Fig. 3).

**Figure 4:**
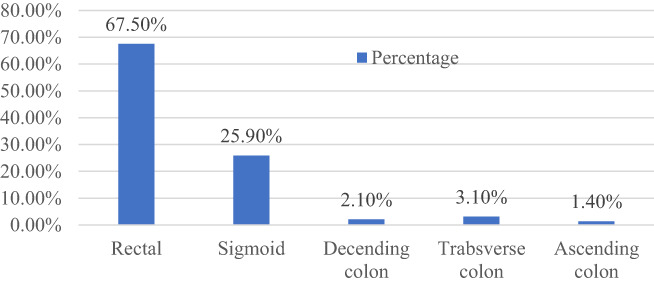
Distribution of colorectal polyps.

According to the location, rectum and sigmoid colon were the common sites of occurrence of polyps, but they were less frequent in other parts of the colon (see Fig. 4).

## Discussion

We investigated the epidemiological profile of pediatric patients who underwent colonoscopy procedures and found hematochezia and constipation were the common indication. Colorectal polyps and inflammation were the most frequent findings, and colonoscopy was performed generally without major complications in these patients. To the best of our knowledge, this is the first study to evaluate colonoscopic findings in children with LGI complaints. No epidemiological data regarding the overall incidence of colonoscopic findings and colorectal polyps exist in Jalalabad, Afghanistan.

In our study, we defined the age criteria for pediatric patients as ≤16 years. Various cut‐offs ranging from 5 years to 20 years have been used in previous studies.^5^, ^6^, ^11^, ^12^, ^13^ The median age in this study was 7.41 years, which was similar to that in some previous reports, but some were also older and younger in these studies. The male/female ratio was 2.5:1, which was consistent with previous studies.^5^, ^6^, ^11^, ^12^, ^13^ However, the reason for this differences in sex have not been understood yet.

We compared positive macroscopic findings (81.2%) with those of other studies of children younger than 16 or 18 years such as from the United Stated, Korea, South China, and Hong Kong, which were 62%, 45.8%, 70.5%, and 50.6%, respectively,^5^, ^14^, ^15^, ^16^ which are lower. Re‐indication by the endoscopist and late reference due to low awareness of doctors in the eastern region might be the reasons for the high rate of positive findings. The cecal intubation rate was 67.2% and ileal intubation rate was 50% in this study. The presence of severe colitis (which has a high risk of complications and was not indicated by the endoscopist), poor bowel preparation, and technical failure were the main reasons of incomplete colonoscopy.

Hematochezia, abdominal pain/discomfort, and diarrhea are reported to be the most common indications in children compared to adults.^5^ In this study, hematochezia, diarrhea, and constipation were the most frequent presentations in children referred for colonoscopy. We found that 80.4% of children who underwent colonoscopy had rectal bleeding as a primary indication. We similarly found that rectal bleeding is the most frequent presentation for colonoscopy in children of all ages.^5^, ^6^, ^11^, ^13^, ^16^, ^17^, ^18^ In one study, hematochezia, abdominal pain/discomfort, and diarrhea were the common presentations, accounting for 48.8%, 41.3%, and 11.8%, respectively.^13^

In our study, the detection rate of colorectal polyps was 50%, with peak age between 5 and 8 years with frequent presentation of hematochezia. In a study based in Hong Kong, the detection rate of colorectal polyps in pediatric patients was found to be 29.1%.^16^ In a large cohort study from 14 medical centers in the United States, the detection rate for colorectal polyps was 61% in pediatric patients.^12^ In another study conducted in south China, the detection rete was 42.9% in pediatric patients.^5^ In a different study, colorectal polyps were found to peak between ages 3 and 6 years, and another study found 88.1% colorectal polyps in 2–7‐year‐olds.^13^, ^17^ The peak ages in the above studies are very similar to that of our study but the prevalence rates are different. In the literature, the prevalence of colorectal polyps greatly varied in pediatric patients who underwent colonoscopy. The difference in detection rate might be due to the difference in colonoscopy indications in each healthcare system, as well as the patients' age and ethnicity. However, in our study, the prevalence of polyps was low in children above 8 years of age (Fig. 1) and was similar to those of other studies. Colorectal polyps have a natural tendency to disappear and spontaneously cure in late childhood.^19^, ^20^

Our study morphologically identified 96% colorectal polyps as pedunculated and 4% as sessile; 75% were solitary, 16.80% double, 2.80% triple, 2.40% quadruple, and 3% multiple. Distribution of polyps along the colon was found to be 67.5%, 25.90%, 2.10%, 3.10%, and 1.40% in the rectum, sigmoid, descending colon, transverse colon, and ascending colon, respectively. In two patients rectal and in one patient anal canal mucosal growth with stricture were found in the 13–16‐year age group. In other studies, the commonly occurring type of polyps was found to be juvenile, which were mostly single and morphologically pedunculated, with the majority located in the rectum and sigmoid colon.^16^, ^21^, ^22^, ^23^, ^24^

From the last few decades, pediatric colonoscopy has been widely used for different clinical states, including the diagnosis and evaluation of gastrointestinal bleeding and IBD as well as pathological tissue diagnosis, but in the recent decade, it is also performed for therapeutic applications. The safety of pediatric colonoscopy has been established, and the immediate complication rate has been reported to be 1.1%, which is, however, higher than in adults (0.3%).^25^, ^26^ Our research found no serious complication during the procedure, such as bleeding and perforation, which was due to the careful and conscientious procedure, good bowel cleansing, sufficient risk evaluation preceding the procedure, and appropriate sedation or anesthesia by the endoscopist. We also demonstrated that colonoscopy is as important in children as in adults.

The lack of histological confirmation of the macroscopic colonoscopic findings was the main limitation of this retrospective study. For pediatric patients presenting with recurrent hematochezia, unrelieved abdominal pain, and/or unexplained diarrhea, colonoscopy may be the most useful diagnostic tool.

## Conclusion

Hematochezia is the main indication for performing colonoscopy in children, in whom the most frequent finding is colorectal polyps, which is common in 2–8‐year‐olds. Also, colonoscopy is an important and safe procedure in children for the diagnosis of LGI tract diseases, especially in those who are suffering from painless bleeding as well as unresponsive bloody and chronic diarrhea.
